# Early childhood education and care-based healthy eating interventions for improving child diet: a systematic review protocol

**DOI:** 10.1186/s13643-020-01440-4

**Published:** 2020-08-13

**Authors:** Jacklyn Jackson, Luke Wolfenden, Alice Grady, Melanie Lum, Alecia Leonard, Sam McCrabb, Alix Hall, Nicole Pearson, Courtney Barnes, Sze Lin Yoong

**Affiliations:** 1 Hunter New England Population Health, Wallsend, New South Wales 2287 Australia; 2 School of Medicine and Public Health, University of Newcastle, Callaghan, New South Wales 2308 Australia; 3 Hunter Medical Research Institute, Newcastle, New South Wales 2300 Australia; 4 Priority Research Centre for Health Behaviour, University of Newcastle, Callaghan, New South Wales 2308 Australia

**Keywords:** Early childhood education and care, Child diet, Healthy eating, Systematic review protocol

## Abstract

**Introduction:**

Diet during infancy and early childhood can have implications on child growth, health, and developmental trajectories. Yet, poor dietary habits are common in young children, who often consume diets that are not aligned with dietary recommendations. Early childhood education and care (ECEC) is a recommended setting to deliver healthy eating interventions as they offer existing infrastructure and access to a large number of children. This protocol aims to describe the methods of a systematic review to assess the effectiveness of healthy eating interventions conducted within the ECEC setting to improve child diet.

**Methods and analysis:**

Eight electronic databases including Cochrane Central Register of Controlled Trials (CENTRAL), Ovid MEDLINE, EMBASE, CINAHL Complete, PsycINFO, ERIC, SCOPUS, and SPORTDiscus will be searched from conception to March 2020. Randomised controlled trials (RCT) of dietary interventions targeting children aged up to 6 years conducted within the ECEC setting (including pre-schools, kindergartens, long day care, and family day care) will be included in the review. The primary review outcome is any measure of child dietary intake. Secondary outcomes include (i) child anthropometrics, (ii) child cognition, (iii) child mental health, (iv) child quality of life, (v) the absolute cost or cost-effectiveness of included interventions, and (vi) any reported adverse effects. Study inclusion, data extraction, and risk of bias assessments will be performed independently by two reviewers. Meta-analyses will be performed if adequate data is available, else review findings will be described narratively.

**Discussion:**

This systematic review seeks to synthesise the effectiveness of healthy eating interventions conducted within the ECEC setting for improving child diet. This review will also seek to describe the effect of ECEC-based healthy eating interventions on a variety of important secondary outcomes (adverse events and cost-effectiveness) that will enhance the public health policy and practice relevance of review findings.

**Systematic review registration:**

PROSPERO [ID CRD42020153188]

## Introduction

Poor dietary intake represents a major modifiable risk factor for chronic diseases including obesity, cardiovascular disease, and some cancers [1, 2]. In particular, low intake of fruits and vegetables, combined with a high intake of energy-dense, nutrient-poor, discretionary foods and beverages (high in salt, added sugars, and/or saturated fat) has been attributed to a range of chronic health conditions [3, 4]. The first years of life are a critical period for establishing good dietary habits, as they have been found to track into adulthood [2], and can influence child growth, general health, and developmental trajectories [5–7]. For example, unhealthy dietary intakes in children as young as 3 years of age have been found to influence cardiovascular disease markers, including obesity, dyslipidaemia, and high blood pressure, which can persist through to adulthood [8–11]. Despite this, international data indicates that in infants and young children, approximately 30% of total daily energy intake comes from discretionary (energy-dense, nutrient-poor) foods, and over 90% of this population are not consuming the recommended serves of vegetables [12, 13].

Early childhood education and care (ECEC) is increasingly acknowledged as a setting that can offer the foundations for lifelong child learning and development [14]. ECEC services are inclusive of regulated care services such as long day care, preschools, nurseries, kindergartens, occasional care, and family day care services that cater for children up to 6 years, prior to compulsory schooling [15]. In high-income countries such as Australia, Canada, the UK, Denmark, Norway, and Germany, approximately 80 to 90% of children aged under 5 years attend some form of ECEC, for an average of up to 30 h per week [16–19]. Given that ECEC services represent a captive setting, where children can consume up to two thirds of their recommended daily intake [20], it represents a promising avenue for targeting dietary behaviours in infants and young children [21–23].

The Determinants of Nutrition and Eating (DONE) framework (2.0) suggests that a range of factors can influence dietary choices [24]. Such determinants include food availability and accessibility, portion sizes, food beliefs and habits, the eating environment, and exposure to food promotion and marketing [24]. Thus, in most countries with regulated ECEC services, licensing and accreditation standards exist to support child wellbeing and healthy eating, by requiring ECEC services to implement practices and policies to support children to consume nutritious foods that help meet the social, cultural, and educational needs of the children [25, 26]. Additional standards related to ECEC nutrition policies, education, and role modelling for ECEC staff are also encouraged [25]. As such, ECEC is a recommended setting for influencing child diet due to its broad reach and available infrastructure to support the delivery of healthy eating interventions.

An umbrella review including 12 systematic reviews by Matwiejczyk and colleagues [27] indicated that most ECEC-based interventions seeking to improve children’s eating habits were typically found to improve at least one measured dietary component including food groups and/or nutrient intakes [27], further suggesting that the most impactful interventions were those focused on environmental changes such as menu modifications, policy, and changes to food provision coupled with technical support and training [27]. It must be highlighted however that these conclusions are based on systematic review searches conducted over 3 years ago and may not reflect updated intervention approaches and findings, given the continued policy and public health commitment to the sector [28, 29]. Therefore, the aim of this systematic review is to undertake an updated systematic literature search to evaluate the effectiveness of ECEC-based healthy eating interventions for improving child diet. This review will report additional relevant policy decision-making outcomes including cost-effectiveness and adverse effects of ECEC-based healthy eating interventions, which have not been previously reported on in other reviews [30].

## Objectives

The primary objective of this systematic review is:

- To describe the effectiveness of ECEC-based healthy eating interventions for improving child dietary outcomes.

Secondary objectives of this systematic review are:

- To describe the effectiveness of ECEC-based healthy eating interventions for improving measures of child weight status
- To describe the effectiveness of ECEC-based healthy eating interventions for improving child cardiovascular disease risk markers
- To describe the effectiveness of ECEC-based healthy eating interventions in improving child cognitive, mental health and quality of life outcomes
- To describe the absolute cost or cost-effectiveness of the included interventions
- To describe any adverse events or unintended effects related to included interventions

## Methods and analysis

This article seeks to describe the methods of a systematic review and meta-analysis according to the Preferred Reporting Items for Systematic Review and Meta-analysis Protocols (PRISMA-P) [31]. The protocol is registered with PROSPERO the international prospective register for systematic reviews, ID CRD42020153188.

### Criteria for considering studies for this review

#### Types of studies

We will include only RCTs (including cluster-RCTs, stepped-wedge RCTs, factorial RCTs, multiple baseline RCTs, and randomised crossover trials). RCTs are considered the highest quality study design for establishing causality, as such we expect this will provide a more accurate estimation of the overall effect of ECEC-based healthy eating interventions.

We will only include cluster-RCTs with a minimum of two intervention sites and two control sites, as per the Effective Practice and Organisation of Care (EPOC) recommendations [32].

#### Types of participants

We will include interventions that seek to improve the dietary intake of children attending an ECEC service, conducted in any country internationally. A variety of participant groups may be included in such trials, including (but not limited to):

- Children aged 6 years and under attending the ECEC service.
- Parents, guardians, or carers of children attending the ECEC service.
- Professionals responsible for the care provided to children attending the ECEC service, including ECEC service directors, educators, volunteers, cooks, or other employed staff.
- Those responsible for the oversight and accreditation of ECEC services, including government authorities, or regulatory agencies, or those with the capacity to influence the nutritional practices of ECEC services, such as those involved in the food supply chain.

Studies targeting children with special needs or clinical conditions (e.g. those with a diagnosed disease or health condition) will be excluded.

#### Types of interventions

This review seeks to include ECEC-based healthy eating interventions conducted within the ECEC setting. This setting includes formal paid care such as preschools, nurseries, long day cares, and kindergartens, as well as family day cares (also known as family child care homes and childminding in which a small group of children is offered care within the educator’s home) that offer care for children up to 6 years, prior to compulsory schooling [33].

Included interventions must seek to influence child diet, but may also include other behavioural components including physical activity and sleep. Included interventions may be single-component or multi-component interventions (i.e. interventions that include more than one strategy to influence child diet). There will be no restriction on intervention duration. Interventions that target both the ECEC service and other settings, such as the home, will be included if the ECEC setting was the primary setting of the intervention.

Interventions that focus specifically on examining malnutrition/malnourishment will be excluded. Obesity management interventions (i.e. those that include only children with overweight or obesity) will also be excluded.

#### Control

We will include studies that report the outcomes of an intervention versus no intervention (control), delayed intervention (wait-list control), usual care, or an alternative intervention that does not seek to influence diet.

#### Types of outcomes

##### Primary outcomes

We will include any measure of child dietary intake. Such measures could include assessments of intake that occur during attendance at childcare or overall dietary intake. Dietary intake may be captured using objective methods including nutritional biomarkers such as doubly labelled water (measure of energy consumption), plate waste audits, or direct observations [34]. Child diet may also be evaluated using subjective methods (e.g. parent-reported dietary intake), such as short diet questions, food frequency questionnaires, food diaries, diet histories, and 24-h recalls. Measures of foods or beverages provided to children, for example, served or listed on childcare menus, but do not assess child intake, will be excluded. Measures of child dietary intake may include, but are not limited to:

- Macronutrient intake (e.g. energy (kJ), fat (g), carbohydrate (g), protein (g), fibre (g)).
- Food group intake (e.g. vegetables (g or serving)).
- Intakes of specific dietary components of interest (e.g. sugar (g), or sugar-sweetened beverage (mL)).
- Percent total energy contribution (e.g. percentage of total energy contributed from discretionary/snack foods).
- Measures of overall diet quality (e.g. diet score measuring the consistency of dietary intakes to dietary guidelines).

##### Secondary outcomes

Measures of child weight status or anthropometric measures could be parent-reported, or measured by trained researchers, or ECEC staff. Specific anthropometric measures of interest include:

- Absolute weight in kilograms (kg)
- Body mass index (BMI)/zBMI score
- Waist circumference (cm)
- Waist-to-hip ratio
- Ponderal index
- Percent body fat
- Skin-fold thickness

Measures of child cardiovascular disease risk markers may include:

- Blood pressure (e.g. systolic blood pressure/diastolic blood pressure)
- Blood lipids (e.g. total cholesterol, LDL cholesterol, Apo B, triglycerides, HDL-cholesterol, Apo A-1)
- Blood glucose (e.g. measure of blood glucose, glucose tolerance test, HbA1c)

Measures of child cognitive performance may include [35], but are not limited to:

- Bayley Scale of Infant Development [36].
- Kaufman Assessment Battery for Children [37].
- Wechsler Preschool and Primary Scale of Intelligence [38].
- Stanford-Binet Intelligence Scale [39].
- Differential Abilities Scales [40].
- The early years toolbox for assessing early executive function, language, self-regulation, and social development [41].

Measures of child mental health may include, but are not limited to:

- The Child Behavioural Checklist (CBCL) [42].

Measures of child quality of life may include, but are not limited to:

- The Paediatric Quality of Life Inventory [43].

Estimates of the intervention absolute cost or assessment of the intervention cost-effectiveness may include:

- Cost-effectiveness ratio
- Cost of the program per year of life saved
- Cost of the intervention for each quality-adjusted life years (QALY) gained

Unintended adverse consequences of the interventions could be assessed using questionnaires, surveys, direct observations, or service audits, and may relate to:

- Child health
- Service operations
- Staff/parent attitudes

### Search methods for identification of studies

We will use a search strategy based on a previously conducted Cochrane review [44], adapted by a research librarian to suit our research question. The search was based on the following domains using Medical Subject Headings (MeSH) for ‘diet/nutrition’ and ‘ECEC’ and ‘randomised controlled trial’ and ‘humans’. Our search terms for each electronic database are outlined in Table S1 [see Additional file 1].

#### Electronic searches

A systematic search strategy will be undertaken from database conception until March 2020 using the following electronic databases:

- Cochrane Central Register of Controlled Trial (CENTRAL);
- Ovid MEDLINE;
- EMBASE;
- CINAHL Complete;
- PsycINFO;
- ERIC;
- SCOPUS;
- SPORTDiscus.

We will not impose any language or time restrictions on the searches.

#### Unpublished or grey literature searches

In addition to electronic database searches, we will search for relevant unpublished or grey literature publications using the following:

- The World Health Organization International Clinical Trials Registry Platform (www.who.int/intrp)
- The registry ClinicalTrials.gov (www.clinical.trials.gov)
- Google Scholar (first 100 results)

#### Searching other resources

Additional searches we will undertake include:

- Hand reference list researches of included studies.
- Authors of relevant protocol papers identified by the electronic database searches will be contacted.

### Data collection and analysis

#### Selection of studies

Pairs of review authors will independently screen titles and abstracts of all studies using Covidence software [45]. If discrepancies between reviewers cannot be resolved by consensus, a third reviewer will be consulted to inform study progression to full-text review. We will contact authors if study information to inform study inclusion is unavailable or unclear.

Full-text articles will be obtained for any study which could not clearly be excluded on the basis of study title and abstract. Full-text articles will be reviewed for their eligibility for inclusion by pairs of review authors. If discrepancies cannot be resolved by consensus, a third reviewer will be consulted to inform study inclusion. Reasons for excluding any full-text manuscripts will be documented at this stage, and we will record the selection process in sufficient detail to complete a PRISMA flow diagram [31].

#### Data extraction and management

Pairs of independent, un-blinded reviewers will extract data for included studies. If discrepancies between reviewers are not resolved by consensus, a third reviewer will be consulted for final decision-making.

For included studies, we will use a piloted and adapted version of the Cochrane Public Health data extraction template to extract data on:

- Study characteristics: first author, publication year, country, study design, sample size, funding source;
- Childcare service characteristics: type (centre-based (preschool or long day care) or family day care), operational characteristics (public or private; full-time or part-time), location (urban or rural);
- Participant characteristics: age, gender, ethnicity;
- Intervention characteristics: name of the programme, intervention description, duration, and intensity of the intervention;
- Outcome definitions and time points of outcome measurement;
- Study results relevant to our review outcomes;
- Dropout/adherence rate;
- Financial cost of the intervention;
- Unintended adverse events of the intervention;
- Conflict of interest, using the Tool for Addressing Conflicts of Interest in Trials (TACIT: http://tacit.one/).

### Assessment of risk of bias

Individual study risk of bias will be independently assessed by two reviewers, using the Cochrane Collaboration’s risk of bias (RoB) tool described in the Cochrane Handbook for Systematic Reviews of Interventions [46]. Where required, a third review author will adjudicate discrepancies regarding RoB that could not be resolved via consensus.

For the purposes of this review, RoB domains of interest will be based on the effect of assignment, i.e. whether the interventions were effective regardless of whether the intervention was received as intended (the intention-to-treat effect) [47]. This was chosen as it is most appropriate for informing health policy questions about which interventions should be recommended.

The specific domains of bias reviewed will relate to:

- Selection bias
- Performance bias
- Detection bias
- Attrition bias
- Reporting bias
- Other bias

For cluster RCT, an additional domain will be assessed related to biases arising from the timing of identification and recruitment of participants [48]. Based on RoB assessment, RoB will be judged as ‘low’, ‘high’, or ‘unclear’ and will be used to summarise individual study results, as well as an overall study RoB [47].

### Measures of treatment effect

If meta-analyses are performed, we will report the intervention effect for binary outcomes using risk ratios (RRs), and for continuous outcomes as the mean difference (MD) or standardised mean difference (SMD) if different measures are used to assess the same outcome. Ninety-five percent confidence intervals (CIs) will be calculated and reported for all estimated intervention effects [46].

#### Unit of analysis issues

We will extract data from trials that allocate either individuals or groups to a diet-focused intervention or control, or alternative non-diet-focused intervention. Data from cluster designed trials will be combined with other study outcome data if clustering has been appropriately accounted for. If clustering has not been accounted for in cluster trial analyses, relevant data including the intra-class correlation coefficient (ICC) and average cluster size will be sought and used to calculate the design effect and effective sample size to allow for inclusion of such trials in any meta-analyses [48].

#### Dealing with missing data

Missing data and dropouts in the included studies will be assessed and reported; this will include reported numbers as well as characteristics and reasons for dropout. The authors of the included studies will be contacted to obtain missing data if required. Evidence of potential reporting bias will be documented in the ‘Risk of Bias’ tables.

#### Assessment of heterogeneity

Heterogeneity may be present in the results of included studies due to differences in intervention types and study outcomes. However, provided that sufficient data is available, we will conduct a meta-analysis to quantify the overall effectiveness of interventions for our primary outcome (i.e. child diet). Heterogeneity will be evaluated using forest plots and examining them for asymmetry. In addition, we will quantify statistical heterogeneity by calculating the *I*^2^ statistic [49]. Study heterogeneity will be informed by a narrative description of study characteristics, and causes for study heterogeneity will be explored by subgroup analyses.

#### Assessment of reporting biases

We will assess reporting bias by comparing published reports with information provided in trial registers and protocols. Reporting bias will be explored in any meta-analyses conducted by plotting contour-enhanced funnel plots and visually assessing them for asymmetry and outliers. Given that small studies are consistently more likely to report positive effects, we will also evaluate the presence of reporting bias or differences in the results between smaller and larger studies.

#### Data synthesis

Provided there is adequate data available, we plan to pool measures of the same quantitative outcomes (primary and/or secondary), if the outcomes are comparable and sufficiently homogenous. If studies report multiple outcome measures relating to the same or a similar outcome being pooled, we will use the outcome measure used in the sample size calculation. If a sample size calculation is missing, the primary outcome will be identified by matching the outcome to the primary study aim. For trials with multiple follow-up periods, we will use outcome data from the final follow-up period reported.

Random effects meta-analyses will be used to calculate pooled effects. It is expected that a mix of change-from-baseline and post-intervention measurements will be reported and included in any meta-analyses, using recommended methods where possible [50].

In all instances where we cannot combine data in a meta-analysis, we will conduct a narrative summary of the trial findings in accordance with the procedures outlined in the Cochrane Handbook. This narrative summary will encompass vote counting based on the direction of intervention effect, as well as summarizing intervention effect estimates where available according to the review objectives [51].

#### GRADE and ‘Summary of findings’ table

Grading of Recommendations, Assessment, Development and Evaluation (GRADE) will be used to assess the overall certainty of the available evidence for our primary outcome as recommended by the Cochrane handbook [52, 53]. These results will be presented in a ‘Summary of findings’ table. Based on our GRADE assessment, we will make decisions regarding our level of certainty that the estimates of the effect are correct. Our level of certainty will be presented as either high, moderate, low, or very low.

As per GRADE recommendations, the primary outcome measure will be assessed against eight GRADE criteria to obtain an overall GRADE rating and provide an overall level of certainty of the evidence. We will consider five criteria for lowering the level of certainty: risk of bias, inconsistency, indirectness, imprecision, and publication bias. Following this, the level of certainty may be raised by three criteria: strong association between intervention and outcome, dose-response relationship, and where plausible confounders would have reduced the effect between intervention and outcome. Decisions to downgrade or upgrade the certainty of the evidence for each criterion will be documented using footnotes.

We will present the results in tables. These tables will also report on the number of included studies and participants, the treatment effect estimate, and the assessment of the overall certainty of the body of evidence for that outcome.

#### Subgroup analysis and investigation of heterogeneity

If significant heterogeneity is present, we will conduct subgroup analyses to explore the possible causes of heterogeneity. Provided sufficient data is available, we will explore heterogeneity across subgroups related to population, intervention, comparison, and outcome (PICO) characteristics.

#### Sensitivity analysis

The impact of the study methodological risk of bias will be explored in a sensitivity analysis. To do this, we will repeat any meta-analyses by excluding data from studies classified as a high risk of bias.

## Discussion

Poor dietary intakes can have implications for child short-term and long-term health status [54]. As the ECEC setting provides significant opportunity to deliver public health interventions to improve child diet, identifying effective healthy eating interventions in this setting is critical for supporting population-wide obesity prevention efforts [55]. This systematic review seeks to provide an up-to-date synthesis of healthy eating interventions in ECEC services and describe their effect on child diet, anthropometrics, cardiovascular disease risk markers, and other related health outcomes including cognition, mental health, and quality of life.

Such a synthesis is particularly important in this sector as identifying effective interventions in this setting remains an ongoing area of global public health interest and investment [21]. Further, recognising the influence of this setting on child health and wellbeing, there has been a rapid progression of the evidence base, with a large number of RCTs being conducted in this setting in recent years. A review of RCTs provides level 1 evidence of causality to support policy and investment decisions [56]. Additionally, this review seeks to describe potential adverse events, and the absolute-cost/cost-effectiveness of ECEC-based healthy eating interventions, to further support the selection of interventions which are effective, safe, and cost-effective to deliver.

Publishing this systematic review protocol is expected to reduce the possibility of duplication and offers transparencies to the methods and processes used. It also allows outcomes to be pre-specified reducing the risk of reporting biases. Thus, any important protocol amendments will be recorded in PROSPERO. The results from the completed systematic review and meta-analysis will be presented at relevant national/international conferences and will be submitted for peer-review journal publication. The results of this systematic review are expected to support public health practitioners and policy decision-makers by summarising the updated evidence base for healthy eating interventions within the ECEC setting.

## Supplementary information

**Additional file 1.** Search strategy.

## Abbreviations

BMI: Body mass index
CBCL: Child Behavioural Checklist
CENTRAL: Cochrane Central Register of Controlled Trials
CI: Confidence intervals
DONE: Determinants of Nutrition and Eating
ECEC: Early childhood education and care
EPOC: Effective Practice and Organisation of Care
GRADE: Grading of Recommendations, Assessment, Development and Evaluation
ICC: Intra-class correlation coefficient
MD: Mean difference
PICO: Participants, intervention, control, outcome
PRISMA: Preferred Reporting Items for Systematic Reviews and Meta-Analyses
RCT: Randomised controlled trial
RoB: Risk of bias
RR: Risk ratios
SMD: Standardised mean difference
TACIT: Tool for Addressing Conflicts of Interest in Trials
QALY: Quality-adjusted life years
