# Supplementary material for: Early childhood education and care-based healthy eating interventions for improving child diet: a systematic review protocol
Source: Syst Rev. 2020 Aug 13;9:181. doi: 10.1186/s13643-020-01440-4 (PMC7425066; doi:10.1186/s13643-020-01440-4)
Supplement: Supplementary file 1 — Additional file 1. Search strategy. [file 13643_2020_1440_MOESM1_ESM.docx]

Additional File 1

**Table S1. Electronic Databases Search Terms**

| **MEDLINE SEARCH** | |
| --- | --- |
| 1 | Exp Diet/ |
| 2 | Nutrition*.mp. |
| 3 | (health* adj2 eat*).mp |
| 4 | Child Nutrition Sciences/ |
| 5 | Exp Fruit/ |
| 6 | Fruit*.mp. |
| 7 | Vegetable/ |
| 8 | Vegetable*.mp. |
| 9 | Fruit and vegetable juice.mp |
| 10 | Canteen.mp. |
| 11 | Food Services/ |
| 12 | Menu*.mp. |
| 13 | (calorie or calories or kilojoule*).mp. |
| 14 | Energy density.mp. |
| 15 | Eating/ |
| 16 | Feeding Behavio?r*.mp |
| 17 | Dietary intake.mp. |
| 18 | Food habits/ |
| 19 | Food/ |
| 20 | Carbonated beverages/ |
| 21 | Soft drink*.mp. |
| 22 | Soda.mp. |
| 23 | Sweetened drink*.mp. |
| 24 | Dietary fats/ |
| 25 | Confectionary.mp. |
| 26 | (school adj2 (lunch* or meal*)).mp. |
| 27 | Menu planning.mp. |
| 28 | Feeding program*.mp. |
| 29 | Food program*.mp. |
| 30 | (nutrition* adj2 program*).mp. |
| 31 | Cafeteria*.mp. |
| 32 | Nutritional status/ |
| 33 | 1 or 2 or 3 or 4 or 5 or 6 or 7 or 8 or 9 or 10 or 11 or 12 or 13 or 14 or 15 or 16 or 17 or 18 or 19 or 20 or 21 or 22 or 23 or 24 or 25 or 26 or 27 or 28 or 29 or 30 or 31 or 32 |
| 34 | Child, Preschool/ |
| 35 | Child Day Care Centers/ |
| 36 | (childcare* or child care*).mp. |
| 37 | (daycare* or day care*).mp. |
| 38 | Early child*.mp. |
| 39 | (nursery or nurseries).mp. |
| 40 | Kinder*.mp. |
| 41 | (family or home base or homebased or in home).mp. |
| 42 | (pre-school* or preschool*).mp |
| 43 | 34 or 35 or 36 or 37 or 38 or 39 or 40 or 41 or 42 |
| 44 | Randomi?ed controlled trial.pt |
| 45 | Controlled clinical trial.pt |
| 46 | Trial*.tw. |
| 47 | Random*ab. |
| 48 | 44 or 45 or 46 or 47 |
| 49 | 33 and 43 and 48 |
| 50 | Exp animal/ |
| 51 | Humans.sh. |
| 52 | 50 not 51 |
| 53 | 49 not 52 |
| **EMBASE SEARCH** | |
| 1 | Exp diet/ |
| 2 | Nutrition*.mp. |
| 3 | Nutrition/ |
| 4 | (health* adj2 eat*).mp. |
| 5 | Child nutrition sciences.mp. |
| 6 | Nutritional science/ |
| 7 | Fruit*.mp. |
| 8 | Fruit/ |
| 9 | “fruit and vegetable juice”/ |
| 10 | Vegetable*.mp. |
| 11 | Vegetable/ |
| 12 | Canteen*.mp. |
| 13 | Food Services.mp. |
| 14 | Catering service/ |
| 15 | Menu*.mp. |
| 16 | (calorie or calories or kilojoule*).mp. |
| 17 | Energy intake.mp. |
| 18 | Caloric intake/ |
| 19 | Energy density.mp. |
| 20 | Eating/ |
| 21 | Feeding behaviu?r*.mp. |
| 22 | Feeding behaviour/ |
| 23 | Dietary intake.mp. |
| 24 | Dietary intake/ |
| 25 | Food habit*.mp. |
| 26 | Food/ |
| 27 | Carbonated beverage/ |
| 28 | Soft drink*.mp. |
| 29 | Soft drink/ |
| 30 | Soda.mp. |
| 31 | Sweetened drink*.mp. |
| 32 | Dietary fats.mp. |
| 33 | Fat intake/ |
| 34 | Confectionary.mp. |
| 35 | (school adj2(lunch* or meal*)).mp. |
| 36 | Menu Planning.mp. |
| 37 | Feeding program*.mp. |
| 38 | Food program*.mp. |
| 39 | (nutrition* adj2 program*).mp. |
| 40 | Cafeteria*.mp. |
| 41 | Nutritional status/ |
| 42 | 1 or 2 or 3 or 4 or 5 or 6 or 7 or 8 or 9 or 10 or 11 or 12 or 13 or 14 or 15 or 16 or 17 or 18 or 19 or 20 or 21 or 22 or 23 or 24 or 25 or 26 or 27 or 28 or 29 or 30 or 31 or 32 or 33 or 34 or 35 or 36 or 37 or 38 or 39 or 40 or 41 |
| 43 | Child, Preschool/ |
| 44 | (pre-school* or preschool*).mp. |
| 45 | Day care/ |
| 46 | Child care/ |
| 47 | Childcare*.mp. |
| 48 | (daycare* or day care*).mp. |
| 49 | Early child*.mp. |
| 50 | (nursery or nurseries).tw. |
| 51 | Kinder*.mp. |
| 52 | (family or home based or homebased or ‘in home”).mp. |
| 53 | 43 or 44 or 45 or 46 or 47 or 48 or 49 or 50 or 51 or 52 |
| 54 | “randomized controlled trial”/ |
| 55 | “controlled clinical trial”/ |
| 56 | Random*.ti,ab,tt. |
| 57 | Trial.ti,tt. |
| 58 | 54 or 55 or 56 or 57 |
| 59 | 42 and 53 and 58 |
| 60 | Animal experiment/ |
| 61 | “human experiment”/ |
| 62 | Human/ |
| 63 | 61 or 62 |
| 64 | 60 not 63 |
| 65 | 59 not 64 |
| **PSYCINFO SEARCH** | |
| 1 | Diets/ |
| 2 | Exp Nutrition/ or Nutrition*.mp. |
| 3 | (health* adj2 eat*).mp. |
| 4 | Child Nutrition Sciences.mp. |
| 5 | Fruit*.mp. |
| 6 | Vegetable*.mp. |
| 7 | Canteen*.mp. |
| 8 | Food Services.mp. |
| 9 | Menu*.mp. |
| 10 | (calorie or calories or kilojoule*).mp. |
| 11 | Food Intake/ or Energy Intake.mp. |
| 12 | Energy density.mp. |
| 13 | Eating.mp. |
| 14 | Eating behaviour/ |
| 15 | Feeding behavio?r*.mp. |
| 16 | Dietary intake.mp. |
| 17 | Food/ |
| 18 | ((carbonated or sweetened or soft) adj (drink* or beverage*)).mp. |
| 19 | Soda.mp. |
| 20 | Dietary fat*.mp. |
| 21 | Confectionary.mp. |
| 22 | (school adj2 (lunch* or meal*)).mp. |
| 23 | Feeding program*.mp. |
| 24 | Food program*.mp. |
| 25 | (nutrition* adj2 program*).mp. |
| 26 | Cafeteria*.mp. |
| 27 | 1 or 2 or 3 or 4 or 5 or 6 or 7 or 8 or 9 or 10 or 11 or 12 or 13 or 14 or 15 or 16 or 17 or 18 or 19 or 20 or 21 or 22 or 23 or 24 or 25 or 26 |
| 28 | Preschool students/ or nursery school students/ |
| 29 | (pre-school* or preschool).mp. |
| 30 | Day Care Centers/ or Child Day Care/ |
| 31 | (childcare* or child care*).mp. |
| 32 | (daycare* or day care*).mp. |
| 33 | Early child*.mp. |
| 34 | (nurseries or nursery).mp. |
| 35 | Kindergarten Students/ or Kinder*.mp. |
| 36 | (family or home based or homebased).mp. |
| 37 | 28 or 29 or 30 or 31 or 32 or 33 or 34 or 35 or 36 |
| 38 | Randomi?ed controlled trial*.mp. |
| 39 | Clinical Trials/ |
| 40 | Trail*.tw. |
| 41 | Random*.ab. |
| 42 | 38 or 39 or 40 or 41 |
| 43 | 27 and 37 or 42 |
| **CINAHL Complete SEARCH** | |
| S48 | S30 and S41 and S46 (limited to humans) |
| S47 | S30 and S41 and S46 |
| S46 | S42 or S43 or S44 or S45 |
| S45 | AB random* |
| S44 | TI trial* or AB trial* |
| S43 | (MH “Clinical Trials”) |
| S42 | (MH “Randomized Controlled Trials”) |
| S41 | S31 or S32 or S33 or S34 or S35 or S36 or S37 or S38 or S39 or S40 |
| S40 | Family or “home based” or homebased or “in home” |
| S39 | Kinder* |
| S38 | Nursery or nurseries* |
| S37 | (MH “Schools, Nursery”) |
| S36 | “early child*” |
| S35 | Daycare* or “day care*” |
| S34 | Childcare* or “child care*” |
| S33 | (MH “child day care”) OR (MH “Child care providers”) OR (MH “child care (Saba CCC)”) OR (MH “ Child Care”) |
| S32 | “pre-school*” or preschool* |
| S31 | (MH “Child, Preschool”) |
| S30 | S1 or S2 or S3 or S4 or S5 or S6 or S7 or S8 or S9 or S10 or S11 or S12 or S13 or S14 or S15 or S16 or S17 or S18 or S19 or S20 or S21 or S22 or S23 or S24 or S25 or S26 or S27 or S28 or S29 |
| S29 | (MH “ Nutritional Status”) |
| S28 | Cafeteria* |
| S27 | (nutrition* n2 program*) |
| S26 | “food program*” |
| S25 | “feeding program*” |
| S24 | School n2 (lunch* or meal*) |
| S23 | “confectionary” OR (MH “candy”) |
| S22 | (MH “Dietary Fats”) |
| S21 | “Sweetened drink*” |
| S20 | Soda |
| S19 | (MH “Carbonated beverages”) OR “soft drink*” |
| S18 | (MH “Food”) |
| S17 | (MH “Food Habits”) |
| S16 | “dietary intake” |
| S15 | (MH “Eating”) OR (MH “Eating Behaviour”) |
| S14 | “feeding behavio?r*” |
| S13 | (MH “Energy Density”) OR “Energy Density” |
| S12 | (MH “Energy Intake”) OR (MH “Food Intake”) |
| S11 | Calorie or calories or kilojoule* |
| S10 | (MH “Menu Planning”) OR “menu*” |
| S9 | (MH “Food Services”) |
| S8 | “canteen*” |
| S7 | Fruit* |
| S6 | (MH “Vegetables”) Or “vegetable*” |
| S5 | (MH “Child nutrition”) |
| S4 | Health* n2 eat* |
| S3 | (MH “Nutrition”) |
| S2 | “nutrition*” |
| S1 | (MH “Diet+”) |
| **COCHRANE CENTRAL REGISTER OF CONTROLLED TRIALS (CENTRAL) SEARCH** | |
| #1 | MeSH descriptor: [Diet] explode all trees |
| #2 | (nutrition*): ti,ab,kw |
| #3 | ((health*near/2 eat*)):ti,ab.kw |
| #4 | MeSH descriptor: [child nutrition sciences] explode all trees |
| #5 | (fruit*):ti,ab,kw |
| #6 | MeSH descriptor: [Fruit] this term only |
| #7 | (vegetable*):ti,ab,kw |
| #8 | MeSH descriptor: [vegetables] this term only |
| #9 | (canteen*):ti,ab,kw |
| #10 | MeSH descriptor: [Fruit and vegetable juice] this term only |
| #11 | MeSH descriptor: [Food Services] this term only |
| #12 | (menu*):ti,ab,kw |
| #13 | (calorie or calories or kilojoule*):ti,ab,kw |
| #14 | (“energy density”): ti,ab,kw |
| #15 | MeSH descriptor: [Eating] this term only |
| #16 | MeSh descriptor: [Feeding Behavior] this term only |
| #17 | (“feeding behavio*”):ti,ab,kw |
| #18 | (“dietary intake”):ti,ab,kw |
| #19 | MeSH descriptor: [Food] this term only |
| #20 | MeSH descriptor: [Carbonated Beverages] this term only |
| #21 | (“soft drink”): ti,ab,kw |
| #22 | (soda):ti,ab,kw |
| #23 | (“sweetened drink*”):ti,ab,kw |
| #24 | MeSH descriptor: [Dietary Fats] this term only |
| #25 | (confectionary):ti,ab,kw |
| #26 | (school near/2 (lunch* or meal*)):ti,ab,lw |
| #27 | MeSH descriptor: [Menu Planning] this term only |
| #28 | (“feeding program*”):ti,ab.kw |
| #29 | (“food program*”):ti,ab,kw |
| #30 | (nutrition* near/2 program*):ti,ab,kw |
| #31 | (cafeteria*):ti,ab,kw |
| #32 | MeSH descriptor: [Nutrition Status] this term only |
| #33 | (49-#32) |
| #34 | MeSH descriptor: [child, preschool] this term only |
| #35 | (“pre-school*” or preschool*):ti,ab,kw |
| #36 | MeSH descriptor: [Child Day Care Centers] this term only |
| #37 | (childcare* or “child care*”):ti,ab,kw |
| #38 | (daycare* or “day care*”):ti,ab,kw |
| #39 | (“early child*”):ti,ab,kw |
| #40 | (nursery or nurseries):ti,ab,kw |
| #41 | (Kinder):ti,ab,kw |
| #42 | Family or homebased or “home based” or “in home” |
| #43 | or # 34-#42 |
| #44 | and # 33, # 43 |
| **SPORTDiscus SEARCH** | |
| S46 | S30 AND S40 AND S45 |
| S45 | S41 OR S42 OR S43 OR S44 |
| S44 | AB trial* or random* |
| S43 | TI trial* |
| S42 | “clinical trials” |
| S41 | Randomised controlled trial or randomized controlled trial or rct |
| S40 | S31 or S32 or S33 or S34 or S35 or S36 or S38 or S39 |
| S39 | Family or “home based” or homebased or “in home” |
| S38 | Kindergarten or preschool or early childhood education |
| S37 | Nursery or nurseries |
| S36 | “early child*” |
| S35 | Daycare* or “day care” |
| S34 | Childcare* |
| S33 | “child day care” or “child care providers” or “child care (saba CCC)” or “child care” |
| S32 | “pre-school*” or preschool* |
| S31 | AB child, preschool |
| S30 | S1 or S2 or S3 or S4 or S5 or S6 or S7 or S8 or S9 or S10 or S11 or S12 or S13 or S14 or S15 or S16 or S17 or S18 or S19 or S20 or S21 or S22 or S23 or S24 or S25 or S26 or S27 or S28 or S29 |
| S29 | (AB “Nutritional Status’) |
| S28 | Cafeteria* |
| S27 | (nutrition* n2 program*) |
| S26 | ”food program*” |
| S25 | “feeding program*” |
| S24 | School n2 (lunch* or meal*) |
| S23 | “confectionary” OR (AB “candy”) |
| S22 | (AB “Dietary Fats”) |
| S21 | “Sweetened drink*” |
| S20 | Soda |
| S19 | (AB “Carbonated beverages” OR “Soft drink*” |
| S18 | (AB “Food”) |
| S17 | (AB “food habits”) |
| S16 | “dietary intake” |
| S15 | (AB “Eating”) OR (AB “Eating Behaviour”) |
| S14 | “feeding behavio?r*” |
| S13 | (AB “Energy density”) OR “Energy density” |
| S12 | (AB “Energy Intake”) OR (AB “Food Intake”) |
| S11 | Calorie or calories or kilojoule* |
| S10 | (AB “Menu Planning”) OR “menu*” |
| S9 | (AB “Food Services”) |
| S8 | “canteen*” |
| S7 | Fruit* |
| S6 | (AB “vegetables” OR “vegetable*” |
| S5 | (AB “Child nutrition”) |
| S4 | Health* n2 eat* |
| S3 | (AB “Nutrition”) |
| S2 | “nutrition*” |
| S1 | “Diet” |
| **ERIC- search anywhere SEARCH** | |
| (Diet OR nutrition* OR (health* AND eat*) OR "Child Nutrition*" OR fruit* OR vegetable* OR canteen* OR menu* OR calorie OR calories OR kilojoule* OR "Energy Intake" OR "energy density" OR Eating OR "Feeding Behavio*" OR "dietary intake" OR food OR ((carbonated OR sweetened OR soft) AND (drink* OR beverage*)) OR soda OR "Dietary Fat*" OR confectionary OR (school AND (lunch* OR meal*)) OR "feeding program*" OR cafeteria*)  AND (“pre-school*” or preschool* or childcare* or “child care*” or daycare* or “day care*” or “early child*” or nursery or nurseries or Kinder* or family or homebased or "home based" or "in home")  AND (Random* or trial*) | |
| **SCOPUS SEARCH** | |
| TITLE-ABS ( diet  OR  nutrition*  OR  ( health*  w/2  eat* )  OR  "Child Nutrition*"  OR  fruit*  OR  vegetable*  OR  canteen*  OR  menu*  OR  calorie  OR  calories  OR  kilojoule*  OR  "Energy Intake"  OR  "energy density"  OR  eating  OR  "Feeding Behavio*"  OR  "dietary intake"  OR  food  OR  ( ( carbonated  OR  sweetened  OR  soft )  AND  ( drink*  OR  beverage* ) )  OR  soda  OR  "Dietary Fat*"  OR  confectionary  OR  ( school  AND  ( lunch*  OR  meal* ) )  OR  "feeding program*"  OR  cafeteria* )  AND  TITLE-ABS ( "pre-school*"  OR  preschool*  OR  childcare*  OR  "child care*"  OR  daycare*  OR  "day care*"  OR  "early child*"  OR  nursery  OR  nurseries  OR  kinder* )  AND  TITLE-ABS ( random*  OR  trial* ) | |
